# The Role of Peroxisome Proliferator-Activated Receptors in Polycystic Ovary Syndrome

**DOI:** 10.3390/jcm12082912

**Published:** 2023-04-17

**Authors:** Iason Psilopatis, Kleio Vrettou, Eleni Nousiopoulou, Kostas Palamaris, Stamatios Theocharis

**Affiliations:** 1 Department of Gynecology, Charité—Universitätsmedizin Berlin, Corporate Member of Freie Universität Berlin and Humboldt—Universität zu Berlin, Augustenburger Platz 1, 13353 Berlin, Germany; 2 First Department of Pathology, Medical School, National and Kapodistrian University of Athens, 75 Mikras Asias Street, Bld 10, Goudi, 11527 Athens, Greece

**Keywords:** peroxisome proliferator-activated receptor, PPAR, polycystic ovary syndrome

## Abstract

Polycystic ovary syndrome (PCOS) constitutes the most common endocrine disorder in women of reproductive age. Patients usually suffer from severe menstrual irregularities, skin conditions, and insulin resistance-associated health conditions. Peroxisome proliferator-activated receptors (PPARs) are nuclear receptor proteins that regulate gene expression. In order to investigate the role of PPARs in the pathophysiology of PCOS, we conducted a literature review using the MEDLINE and LIVIVO databases and were able to identify 74 relevant studies published between 2003 and 2023. Different study groups reached contradictory conclusions in terms of PPAR expression in PCOS. Interestingly, numerous natural agents were found to represent a novel, potent anti-PCOS treatment alternatives. In conclusion, PPARs seem to play a significant role in PCOS.

## 1. Introduction

Polycystic ovary syndrome (PCOS) represents the most common endocrine disorder in women of reproductive age worldwide [1]. The onset of the symptomatology typically occurs during adolescence, with the symptoms ranging from menstrual irregularities and skin conditions to insulin resistance and associated health conditions [2]. In adults, PCOS is diagnosed based on the presence of at least two of the Rotterdam criteria, after the exclusion of other endocrinological conditions. The Rotterdam criteria require the presence of two of the following: oligoovulation and/or anovulation, hyperandrogenism, or enlarged (ovarian volume ≥ 10 mL) and/or polycystic ovary (presence of multiple cystic follicles in one or both ovaries) on ultrasound [3]. Apart from a detailed medical history and a scholastic clinical examination, laboratory studies are essential for the confirmation of hyperandrogenism, as well as the exclusion of differential endocrinological conditions such as hyperprolactinemia or thyroid dysfunction [4]. Given that women with PCOS are at high risk of serious comorbidities, metabolic screening, and monitoring, alongside the evaluation of mental health and quality of life, should take place both at the first visit and at regular time intervals [5]. The standard therapeutic approach for all PCOS patients should always include lifestyle modifications. More precisely, weight reduction, caloric intake restriction, tailored diets, as well as exercise and physical activity, constitute the main pillars of the non-pharmacological approaches [6,7,8]. The pharmacological approach for PCOS patients not wishing to conceive primarily focuses on the control of menstrual cycle abnormalities and hyperandrogenism, the treatment of comorbidities, as well as the improvement of the quality of life. Combined oral contraceptives or progestins represent the first-line drugs of choice for hyperandrogenism and/or menstrual irregularities, while biguanides may be added to the combined oral contraceptives and lifestyle modifications to improve menstrual cycle abnormalities, metabolic outcomes, and weight [9]. Women unable to tolerate combined oral contraceptives may be treated with antiadrogens for treatment of hirsutism and alopecia [9]. PCOS patients planning to conceive should be treated with letrozole or clomiphene in order to induce ovulation [10].

Even though a part of the involved mechanisms in PCOS occurrence is discovered, the exact etiology and pathophysiology are, to date, not well comprehended [11]. External risk factors include epigenetic mechanisms such as gene promoter hyper-/hypomethylations, endocrine-disrupting chemicals, physical and emotional stress, as well as nutrient levels [9]. Insulin resistance, hyperandrogenism, inflammation, oxidative stress, and obesity, summarize the most representative internal molecular mechanisms of PCOS pathogenesis [12]. More accurately, androgen hypersecretion caused by intrinsic dysfunction of theca cells and/or the hypothalamus-pituitary-ovarian axis results in both abnormal gonadotropin-releasing hormone (GnRH) pulsation and gonadotropin secretion through the aberrant negative or positive feedback of female sex hormones [13,14]. The aforementioned abnormal gonadotropin secretion correlates with a high luteinizing hormone (LH)/follicle-stimulating hormone (FSH) ratio, which further provokes ovarian dysfunction and the hypersecretion of androgens [2,15]. Furthermore, anti-Müllerian hormone (AMH) is intensively secreted by the pre−/small antral follicles accumulating in polycystic ovaries, thus inducing deleterious effects on the follicular microenvironment and/or GnRH pulsation [12]. Notably, insulin resistance, and consequently hyperinsulinemia, also intensify androgen secretion by theca cells and inhibit the hepatic production of sex hormone-binding globulin (SHBG), hence augmenting the circulating concentration of bioactive free testosterone [16,17,18]. The described insulin resistance primarily develops in the liver or muscles and correlates with visceral adiposity and adipocyte dysfunction, which are in return aggravated by hyperandrogenism [2]. All in all, the pathology of PCOS may be described as a vicious cycle of complex and heterogenous disorders, with hyperandrogenism constituting a predisposing factor and insulin resistance triggering the development of this complicated syndrome [12].

Peroxisome proliferator-activated receptors (PPARs) represent fatty acid-activated nuclear receptors comprised of three isoforms with discrete metabolic regulatory activities, tissue distribution, and ligand-binding properties: α, β/δ, and γ [19,20,21]. By binding as heterodimers with the retinoid X receptors (RXRs) to specific DNA response elements within promoters, PPARs embody ligand-regulated transcription factors that efficiently promote or inhibit the expression of their target genes [22]. The size of the PPAR ligand binding cavity is significantly larger than that of other nuclear receptors, thereby enabling the attachment of numerous natural and synthetic ligands, which may trigger an exchange of co-repressors for co-activators and stimulate the functions of PPARs [23,24,25,26,27,28,29,30,31,32,33]. PPARs are involved in fatty acid disposition and metabolism, energy homeostasis, cell differentiation, diverse cellular biology functions, as well as immunity mechanisms [34,35]. More specifically, PPARα shows high expression in the heart, liver, intestine, kidneys, skeletal muscles, and brown adipose tissue, and influences fatty acid metabolism. PPARβ/δ is expressed ubiquitously and impacts fatty acid oxidation, as well as the regulation of blood cholesterol and glucose levels. The PPARγ isoform displays the highest expression in adipocytes and significantly contributes to adipogenesis, lipid biosynthesis, and lipoprotein metabolism, aside from insulin sensitivity [23,36].

Importantly, PPAR isotypes have been described to have a significant impact on the metabolic syndrome, given that they co-determine nonalcoholic steatohepatitis progression by regulating liver metabolism, inflammation, and fibrosis, bridge trace elements and metabolic homeostasis, as well as mediate diabetic cardiomyopathy-related molecular effects [37,38,39]. The prevalence of the metabolic syndrome, which by definition includes central obesity, hypertension, insulin resistance, and atherogenic dyslipidemia, is as high as 43% in adult women suffering from PCOS [11]. Accordingly, the clinical features of PCOS may be classified as metabolic syndrome [11]. Moreover, a recent review focused on PCOS and infertility only [40]. In this regard, the purpose of the present literature review is to investigate the role of PPARs in the pathophysiology of PCOS, closely examine their implication in different molecular mechanisms associated with PCOS genesis and progression, as well as explore the feasibility of PPAR targeting for the treatment of PCOS.

## 2. Methodology

### 2.1. Search Strategy

The literature review was conducted using the MEDLINE and LIVIVO databases. 

Publications published between 1999 and 2023 were reviewed. The search was performed by searching first the MEDLINE and then the LIVIVO database for the terms (“peroxisome proliferator-activated receptor” OR “PPAR”) AND “polycystic ovarian syndrome”. The retrieved articles were read and either included or discarded, based on the inclusion criteria. In addition, the bibliographies/reference lists of all selected articles were manually searched.

### 2.2. Inclusion and Exclusion Criteria

Solely original research articles and scientific abstracts written in the English language, that explicitly reported on the role of PPARs in PCOS pathogenesis were included in the data analysis. Studies emphasizing the involvement of PPARs in endocrinological or metabolic disorders other than PCOS (e.g., polycystic kidney syndrome) were excluded. Review articles or original research works written in other languages were not taken into consideration.

### 2.3. Data Extraction

**Figure 1:**
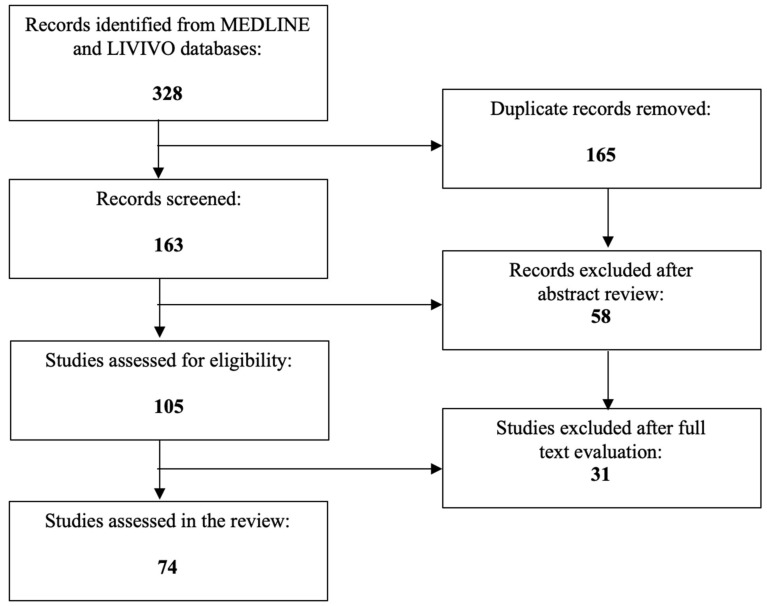
PRISMA flow diagram visually summarizing the screening process.

After the exclusion of duplicates, a total of 163 articles published between 1999 and 2023 were identified. 58 works were discarded in the initial selection process after abstract review. The full texts of the remaining 105 publications were evaluated, and after detailed analysis, a total of 74 relevant studies published between 2003 and 2023, that met the inclusion criteria, were selected for the literature review. Figure 1 presents an overview of the aforementioned selection process.

## 3. PPAR Expression in PCOS (-Induced) Animal Models and Patients

Different research groups have explored the expression of the PPAR isoforms in PCOS.

### 3.1. PPAR

Bai et al. constructed a competitive endogenous RNA (ceRNA) network in PCOS driven by exosomal long non-coding RNA (lncRNA) and, by performing enrichment analysis, concluded that PCOS is mainly enriched in the PPAR signaling pathway [41].

### 3.2. PPARα

In the context of PPARα expression, Morsy et al. treated PCOS-induced rats with the PPARα agonist fenofibrate which significantly increased superoxide dismutase activity, but decreased body weight, as well as serum testosterone, insulin, anti-Mullerian hormone, and ovarian malondialdehyde levels [42]. Furthermore, Tao et al. isolated granulosa cells from the ovaries of PCOS patients and determined that human chorionic gonadotropin and adiponectin significantly upregulate PPARα mRNA and protein expression [43].

### 3.3. PPARγ

In terms of PPARγ and its coactivator-1α (PGC-1α) expression, Amalfi et al. prenatally injected pregnant Sprague Dawley rats with increasing doses of free testosterone, which resulted in different phenotypes of PCOS during the adult life of their female offspring. Of note, evaluation by Western blotting revealed enhanced PPARγ protein expression after administration of higher testosterone doses [44]. However, Emidio et al. used a PCOS mouse model induced by the administration of dehydroepiandrosterone (DHEA) and reported low PGC-1α levels [45], while El-Saka et al. induced PCOS in female Wistar rats by letrozole and underlined the suppression of PPARγ pathways [46]. Cao et al. extracted ovarian granulosa cells from five patients with PCOS and detected a significantly lower expression level of PPARγ mRNA by reverse transcription quantitative real-time polymerase chain reaction (RT-qPCR) than in the control group [47]. Additionally, Lee et al. designated significant PPARγ mRNA downregulation in the granulosa cells of PCOS patients [48] and Qu et al. described lower PPARγ1 mRNA levels in granulosa cells of both hyperandrogenism PCOS patients with failed pregnancies and PCOS-induced rats, with hypermethylated CpG sites in the *PPARγ1* promoter [49]. Skov et al. proved that reduced levels of PGC-1α seemingly contribute to the downregulation of mitochondrial oxidative phosphorylation genes in PCOS [50], whereas Liu et al. investigated the effect of PGC-1α on granulosa cell injury and observed a significant decrease in the expression of PGC-1α, along with a significant upregulation of cell apoptosis and reactive oxygen species (ROS) generation in the obese PCOS patient group [51]. In addition, Hu et al. studied the expression of fatty acid binding protein (FABP4) mRNA in granulosa cells of PCOS women and identified significant upregulation of the *FABP4* gene, which possesses PPARγ response elements in proximal promoter regions [52]. On the contrary, Jansen et al. identified PPARγ as upregulated in PCOS ovaries [53], while Kohan et al. described significantly higher PPARγ gene and protein levels, accompanied by decreased solute carrier family 2 member 4 (SLC2A4) levels, in endometrial tissue of women with PCOS and hyperinsulinemia [54]. Zhao et al. suggested *PGC-1α* promoter methylation and mitochondrial content as predictive biomarkers for metabolic risk in PCOS patients [55]. Last but not least, He et al. treated PCOS and non-PCOS patients with different doses of testosterone and described significantly elevated PPARγ mRNA and protein levels in the experimental group, with PPARγ positively correlating with testosterone concentration [56]. 

Altogether, there seems to be no unanimity on the expression of PPAR isoforms in PCOS subjects. The differences in study design, methodology, sample size, and/or statistics, could have contributed to the distinct results of these studies.

## 4. The Role of *PPAR* Polymorphisms in PCOS

A great number of studies focus on the role of diverse *PPAR* polymorphisms in PCOS patients.

### 4.1. PPAR

Christopoulos et al. estimated and compared the genetic, clinical, hormonal, and metabolic characteristics of 183 PCOS women and 148 healthy volunteers and observed that the *PPARγ* gene polymorphisms do not increase the risk for PCOS (apart from the reduced testosterone levels), whereas the +294T/C polymorphism in the exon 4 of the *PPARδ* gene led to the elevation of fasting glucose levels [57].

### 4.2. PPARγ

Knebel et al. performed sequence analyses of the *PPARγ* gene and denoted that no polymorphism revealed evidence for a direct correlation with the altered interleukin (IL)-7, IL-1β, IL-6, and TNFα levels in PCOS women [58]. Furthermore, Antoine et al. explored the relationship of the *PPARγ* Pro12Ala and silent exon 6 (His447His) polymorphisms with the clinical features of PCOS and stated that Pro12Ala and His447His did not seem to increase the risk of PCOS or its component phenotypes in PCOS patients [59]. In 2003, Orio et al. examined *PPARγ* polymorphisms at exons 2 and 6 in 100 PCOS women and suggested a higher frequency of the C to T substitution in exon 6 of obese PCOS patients, with the Pro12Ala polymorphism at exon 2, however, not affecting body mass index (BMI) in PCOS women [60]. One year later, the same research group confirmed that adiponectin concentrations do not differ between PCOS and controls, with no effect of the Pro12Ala polymorphism on serum adiponectin levels [61]. Xita et al. could also not identify any differences in the distribution of the Pro12Ala polymorphism between PCOS and controls [62]. On the contrary, Zaki et al. described a significant association of the Pro12Ala polymorphism with the risk of PCOS and abnormal metabolic parameters such as BMI, insulin levels, fasting triglycerides, etc. [63]. In 2005, Yilmaz et al. examined the relationship between the Pro12Ala polymorphism and insulin resistance in relatives of PCOS patients and suggested that this gene polymorphism protects against insulin resistance, thereby preventing the development of diabetes mellitus in the first-degree relatives [64]. A year later, the same study group concluded that the same polymorphism may also represent a modifier of insulin resistance in PCOS patients [65]. In 2011, Bidzińska-Speichert et al. performed genetic studies to detect *PPARγ2* Pro12Ala and Pro115Gln gene polymorphism in 54 PCOS patients and reported the absence of the Pro115Gln polymorphism, alongside an estimated frequency of 23.15% in PCOS patients. In this context, BMI ≥ 30 significantly correlated with a higher occurrence of the Ala allele [66]. One year later, the same study group carried out genetic studies to detect the *PPARγ* Pro12Ala gene polymorphism and suggested higher leptin levels in PCOS patients carrying the Pro12Ala genotype than in those with Pro12Pro and Ala12Ala [67]. Tok et al. also noted that PCOS patients with Pro12Ala polymorphism are more obese, have lower fasting insulin levels, and are less insulin-resistant and glucose-intolerant [68]. Moreover, Hahn et al. analyzed *PPARγ* alleles in 102 PCOS patients and 104 age-matched control women and concluded that the Pro12Ala polymorphism correlates with higher insulin sensitivity and decreased hirsutism scores in PCOS women [69], while Koika et al. suggested that the Pro12Ala polymorphism in the *PPARγ2* gene correlates with reduced basic metabolic rate in patients with PCOS and laboratory hyperandrogenemia [70]. Korhonen et al. noted a significant reduction in the frequency of the variant Ala isoform in PCOS patients, accordingly [71]. Rahimi et al. found the *PPARγ* Pro12Ala to be associated with the risk of PCOS and its variant CG genotype to correlate with a lower concentration of estradiol and higher triglyceride levels [72]. Additionally, Shi et al. suggested a significantly higher expression of PPARγ splice variants in PCOS patients, alongside more profound clinical features [73], while Giandalia et al. evaluated 53 PCOS patients and 26 control women and underlined the similar distribution of *PPARγ* exon 2 and exon 6 variants in the two groups. Importantly, *PPARγ* exon 2 and exon 6 variants correlated with differences in the hormonal (17-β estradiol, free testosterone levels) and/or metabolic profile of women with PCOS, thereby indicating their protective effect on insulin resistance and β-cell function [74]. Reddy et al. genotyped three polymorphisms of the *PGC-1α* gene and indicated that *PGC-1α* rs8192678 ‘Ser’ allele carriers are at a higher risk to develop PCOS [75]. 

Taken together, different studies have shown contradictory results concerning the role of *PPAR* polymorphisms in PCOS.

### 4.3. PPAR Polymorphisms Depending on the Ethnic Background

Certain research groups have examined the aforementioned polymorphisms with a special emphasis on the ethnicity of the included study population. 

Baldani et al. enrolled 151 PCOS patients and performed a molecular analysis for the genetic polymorphism which defined the *PPARγ* Pro12Ala polymorphism as a non-significant determinant of PCOS in the Croatian population, with a positive effect on insulin sensitivity and BMI [76]. Chae et al. performed genetic analyses of the *PPARγ* Pro12Ala and the *PGC-1α* Gly482Ser polymorphisms in 184 PCOS patients, but could not identify them as susceptible genes in Korean women suffering from PCOS. Nevertheless, *PPARγ* Pro12Ala polymorphism modulated the concentration of serum high-density lipoprotein (HDL) levels, whereas *PGC-1α* Gly482Ser polymorphism influenced postprandial 2-h insulin levels, accordingly [77]. On the contrary, Gu et al. demonstrated that both Pro12Ala and His447His polymorphisms of *PPARγ* correlate with PCOS in a Korean population [78]. Wang et al. could not find statistically significant differences between *PPARγ2* Pro12Ala and *PGC-1α* Gly482Ser polymorphism distributions between Chinese women with PCOS and controls [79], while Yang et al. also reported no significant difference concerning the *PPARγ2* Pro12Ala polymorphism distributions between Chinese women with PCOS and controls [80]. Dasgupta et al. sequenced 250 PCOS women and 299 controls for *PPARγ* exon 2 and 6 in order to identify distinct single nucleotide polymorphisms in these exonic regions specific to the South Indian population and remarked that the *PPARγ* exon 2 Ala allele and exon 6 His447His T allele were significantly more in the controls than in the PCOS population. In comparison with the haplotypes with wild-type alleles, *PPARγ* haplotypes with mutations depicted a reduced frequency of hyperandrogenic and metabolic features in PCOS [81]. Shaikh et al. investigated the associations of Pro12Ala and His447His *PPARγ* polymorphisms with PCOS susceptibility in an Indian population and concluded that the Pro12Ala polymorphism significantly correlates with diminished PCOS susceptibility, while both polymorphisms improve glucose metabolism by influencing 2 h glucose, fasting insulin, or insulin resistance [82]. Nonetheless, Thangavelu et al. carried out a hospital-based, observational case–control study on PCOS and control Indian women, but the phenotypic variables failed to show any significant difference in the functional single nucleotide polymorphism rs3856806, which is located in exon 6 of *PPARγ* [83]. Table 1 briefly summarizes the aforementioned study results.

Altogether, even in the same ethnic groups, *PPAR* polymorphisms do not seem to necessarily correlate with an increased risk for the presence of PCOS.

## 5. PPAR Expression in PCOS Organ Tissues

To date, several studies have been published on the differential PPAR expression in PCOS cardiac, skeletal, or adipose tissue.

### 5.1. Cardiac Tissue

Tepavčević et al. generated a PCOS-induced rat model that observed an elevation of nuclear PPARα and PGC-1 in cardiac cells [84].

### 5.2. Skeletal Tissue

Dantas et al. published the results of their study incorporating 4 obese PCOS patients, who, after an overnight fast, underwent aerobic exercise. At baseline, *PPARα* and *PGC-1α* were significantly upregulated in the skeletal muscles of PCOS [85]. 

### 5.3. Adipose Tissue

Keller et al. investigated whether adipocyte morphology and gene expression in subcutaneous abdominal adipose differ between late reproductive-aged PCOS-like, prenatally androgenized female monkeys and age-matched controls and reported comparable gene expression of *PPARδ* and *PPARγ* between the two groups [86]. However, Wang et al. described that PPARγ mRNA and protein levels are seemingly low in the adipose tissue of PCOS-induced rats [87], whereas Nada et al. determined changes in the PPARγ mRNA expression in prenatal testosterone-treated sheep (the metabolic characteristics of which resemble the ones of PCOS women) and revealed low *PPARγ* levels in the liver, but high *PPARγ* levels in the adipose tissue [88]. Similarly, Siemienowicz et al. used the same sheep model and reported decreased *PGC-1α*/*PPARγ* expression in the liver and the subcutaneous adipose tissue, accordingly [89].

Maxel et al. measured expression levels of *PPARγ* in subcutaneous adipose tissue of 36 PCOS patients and reported both downregulations with increasing BMI and a positive correlation with the *ZIP14* gene [90]. Moreover, Tao et al. found the PPARγ mRNA expression levels in subcutaneous adipose tissue to be significantly lower in PCOS patients than in BMI-matched controls [91] and Wang et al. noted significant PPARγ downregulation in PCOS subcutaneous adipose tissue [92]. In 2008, Mlinar et al. identified a positive correlation of lipin 1β expression in subcutaneous adipose tissue with *PPARγ* [93], while, three years later, the same study group also identified a positive correlation of *hydroxysteroid 11-beta dehydrogenase 1* (*HSD11B1*) expression in visceral adipose tissue with *PPARγ* [94]. Last but not least, Dumesic et al. cultured subcutaneous abdominal adipose stem cells of PCOS women, which differentiated into adipocytes in vitro without androgen exposure, and found that *PPARγ* gene expression positively predicted total body mass, total body fat, as well as gynoid fat masses [95]. Table 2 provides a brief overview of the above study results.

All in all, PPARγ expression varies depending on each examined PCOS organ tissue.

## 6. The Influence of Natural Agents on PPAR Expression in PCOS

Various study groups have, so far, investigated the effects of natural agents on PPAR expression in PCOS.

### 6.1. PPARα

Kokabiyan et al. treated estradiol valerate-induced PCOS rats with the phenolic component of clove oil eugenol, which significantly boosted *PPARα* gene expression [96]. Additionally, Hai et al. explored the effects of the main component of the Chinese herb Epimedium icariin in rats with PCOS and reported upregulated PPARα mRNA and protein expression, which promotes hepatic mitochondrial fatty acid oxidation and, hence, contributes to the reduction of non-alcoholic fatty liver disease [97]. 

### 6.2. PPARγ

Mansor et al. employed a rat model of induced PCOS and revealed that Labisia pumila standardized water extract augments PPARγ mRNA and protein level expression, as well as enhances the effect of glucose uptake in insulin-resistant adipocytes [98]. Furthermore, Prabhu et al. induced PCOS in female Wistar rats and noted increased PPARγ expression after γ-linolenic acid treatment [99], while Suriyakalaa et al. treated PCOS-induced rats with the fresh leaves extracts of Ficus religiosa, which led to the upregulation of *PPARγ* gene expression [100]. Wen et al. also induced PCOS in female Sprague-Dawley rats and reported activated PPARγ signaling after astragaloside IV treatment [101]. Furthermore, Zhang et al. generated an insulin-resistant PCOS rat model and revealed significant PPARγ upregulation after myoinositol supplementation [102], whereas Safaei et al. examined the effect of vitamin D3 on mitochondrial biogenesis of granulosa cells in a PCOS-induced mouse model and outlined *PGC-1a* upregulation upon vitamin D3 administration [103]. Zaree et al. stated that stimulated follicle-stimulating hormone (FSH)-induced *PPARγ* activity in PCOS granulosa cells suppresses the *CYP-19* gene expression in response to eicosapentaenoic acid administration [104]. Moreover, Nasri et al. conducted a randomized double-blind, placebo-controlled trial among 60 PCOS women and underlined that ω-3 fatty acids supplementation upregulated PPARγ mRNA in peripheral blood mononuclear cells [105]. In 2018, Jamilian et al. allocated 40 PCOS women into two groups and treated them with omega-3 fatty acids plus vitamin E supplements or a placebo and reported upregulated *PPARγ* expression in peripheral blood mononuclear cells of PCOS patients [106]. Two years later, the same research group launched a second randomized, double-blind, placebo-controlled trial to evaluate the effect of curcumin in women suffering from PCOS and revealed *PPARγ* upregulation after curcumin administration [107]. Analogously, Heshmati et al. carried out a randomized placebo-controlled clinical trial and treated 36 PCOS patients with the biologically active phytochemical ingredient curcumin, which significantly increased gene expression of *PGC-1a* [108]. In a third randomized, double-blind, placebo-controlled clinical trial, Jamilian et al. randomly assigned 54 PCOS women to receive either chromium and carnitine co-supplementation or placebo and again highlighted that the above co-supplementation increased *PPARγ* gene expression [109]. Comparably, Amiri Siavashani et al. recruited 40 PCOS patients, who had been selected for in vitro fertilization, in a randomized double-blinded, placebo-controlled trial and discovered that chromium supplementation significantly upregulated *PPARγ* gene expression [110]. Zadeh Modarres et al. conducted a randomized double-blind, placebo-controlled trial among forty infertile PCOS women candidates for in vitro fertilization and reported that selenium supplementation significantly increases *PPARγ* expression levels [111]. In 2018, Rahmani et al. conducted a randomized double-blind, placebo-controlled trial on 40 PCOS patients and described that the coenzyme Q10 upregulates *PPARγ* gene expression in peripheral blood mononuclear cells [112]. The same year, the same researchers conducted a second similar clinical study and demonstrated that fish oil supplementation increases *PPARγ* gene expression, respectively [113]. Shabani et al. performed a randomized, double-blinded, placebo-controlled clinical trial on 58 PCOS women and stated that melatonin supplementation significantly augmented *PPARγ* gene expression [114]. Lastly, Shokrpour et al. conducted a randomized controlled trial on 53 women with PCOS and found that myoinositol supplementation significantly boosted *PPARγ* gene expression [115]. Table 3 briefly summarizes the aforementioned study results. 

Taken together, multiple natural agents seem to potentially influence PPAR expression in PCOS.

## 7. Discussion

PPARs are expressed in ovaries, play an important role in the female reproductive tract and influence fertility [116]. PPARα mRNA is mainly expressed in the ovarian theca and stroma cells, PPARβ/δ mRNA is detected in the whole ovary, whereas PPARγ mRNA is restricted predominantly to granulosa cells in evolving follicles during pseudopregnancy and the estrous cycle [117]. More precisely, PPARγ expression is activated upon folliculogenesis during the early follicle stages, leads to the large follicle stage, and is downregulated following the LH surge [118,119]. As far as the connection between PPARs and PCOS is concerned, Przybycień et al. have recently reported on the role of PPAR ligands such as thiazolidinediones in PCOS, with a special focus on their link with the endocannabinoid system [40]. Nonetheless, no review of the literature has, to date, been published on the exclusive role of the PPAR isoforms in the pathophysiology of PCOS. The present work represents, to our knowledge, the most comprehensive up-to-date literature review, that summarizes the results of all relevant original research studies, and describes the PPAR expression patterns, the *PPAR* polymorphisms, as well as the effect of natural agents on PPAR expression in PCOS. 

The vast majority of researchers have investigated the expression levels of PPARγ (and its coactivator PGC-1α) in PCOS women. Even though some study groups endorsed the assumption that PPARγ expression does not differ between PCOS patients and controls, certain researchers reported significant upregulation, whereas several study teams suggested significantly lower expression levels. Respectively, different study groups published similar contradictory results concerning the distribution of the Pro12Ala polymorphism between PCOS and controls. In the same context, *PPARγ* gene polymorphisms were questionably found to correlate with clinical and/or metabolic features of PCOS women. Remarkably, genomic investigations in women of different ethnic populations also revealed varying, and again contradictory, results in terms of *PPAR* polymorphisms. Moreover, PPARγ expression varies depending on each examined PCOS organ tissue, with diverse study groups describing opposing results concerning the PPAR expression in visceral and/or subcutaneous adipocytes. Taken together, these inconsistencies highlight the necessity for further and more consistent research in this field, with future trials requiring larger patient collectives and scholastic controls for eventual biases such as the ethnic background. Additionally, the unanimous employment of female Wistar rats, alongside standard methods for PCOS induction and common classification criteria (e.g., Rotterdam criteria), could potentially lead to more uniform observations. Except for PPARγ, future studies could also closely investigate the underrated role of the other two PPAR isoforms in PCOS.

Impressively, a great number of studies have explored the effects of natural agents/phytopharmaceuticals on PPAR expression in PCOS. Most research groups are either Iranian, Chinese, or Indian, which may be justified by the fact that Eastern cultures have a long tradition in alternative medicine. As current standard pharmaceutical treatment for PCOS is only symptomatic, but not curative, natural agents could represent great therapeutic alternatives for patients who suffer from severe PCOS symptoms but remain skeptical about standard hormonal therapy. 

One limitation of the present review is the nonsystematic methodology in the context of study selection. Although systematic literature reviews provide the most accurate strategy for the detection of relevant research works, respecting rigorous rules and standards, this approach necessitates a narrow research question that does not cover broader topics, such as the role of PPARs in PCOS. An additional limitation is the eventual evidence selection bias, arising from publication bias, as data from statistically significant studies are more likely to reach publication. Furthermore, the literature analysis was performed by a single person and conducted using two databases. Last but not least, potentially interesting/relevant original research articles not written in the English language had to be excluded. 

## 8. Conclusions

The present review highlights the crucial role of PPARs in PCOS and paves the way for future research directions. Different study groups reached contradictory conclusions in the context of PPAR expression in PCOS. Importantly, various natural agents were identified as a novel, potent anti-PCOS treatment alternatives. In conclusion, PPARs seem to play a significant role in PCOS. Nevertheless, systematic research is further required in this field, in order to reach safe and reproducible results and to comprehensively define the role of PPARs in PCOS pathogenesis and therapy.

## Figures and Tables

**Table 1:** The role of *PPARγ* polymorphisms in PCOS, with a special focus on patients’ ethnicity.

Study	Ethnic Group (Study Group Size)	*PPARγ* Polymorphism	Role in PCOS
Baldani et al. [76]	Croatian (330)	Pro12Ala	Positive effect on insulin sensitivity and BMI
Chae et al. [77]	Korean (440)	Pro12Ala	Modulation of HDL levels
Gu et al. [78]	Korean (238)	Pro12Ala, His447His	Correlation with PCOS
Wang et al. [79]	Chinese (348)	Pro12Ala	No significant correlation
Yang et al. [80]	Chinese (238)	Pro12Ala	No significant correlation
Dasgupta et al. [81]	South Indian (549)	Exon 2 Ala allele, Exon 6 His447His T allele	Reduced frequency of hyperandrogenic and metabolic characteristics
Shaikh et al. [82]	Indian (750)	Pro12Ala, His447His	Improved glucose metabolism, fasting insulin, and insulin resistance
Thangavelu et al. [83]	Indian (338)	rs3856806	No significant correlation

**Table 2:** PPAR expression in PCOS organ tissues.

Study	Study Group Size	PCOS Organ Tissue	PPAR Expression
Tepavčević et al. [84]	24 rats	Cardiac cells	Enhanced nuclear PPARα and PGC-1 expression
Dantas et al. [85]	8 women	Skeletal myocytes	Significant *PPARα* and *PGC-1α* upregulation
Keller et al. [86]	12 monkeys	Subcutaneous adipocytes	No significant differences
Wang et al. [87]	16 rats	Adipocytes	Low PPARγ expression levels
Nada et al. [88]	13 sheep	Hepatocytes, Adipocytes	Low *PPARγ* levels in the liver, High *PPARγ* levels in the adipose tissue
Siemienowicz et al. [89]	121 ewes/lamps	Hepatocytes, Subcutaneous adipocytes	Decreased *PGC-1α*/*PPARγ* expression
Maxel et al. [90]	59 women	Subcutaneous adipocytes	Downregulation with increasing BMI, Positive correlation with the *ZIP14* gene
Tao et al. [91]	34 women	Subcutaneous adipocytes	Low PPARγ expression levels
Wang et al. [92]	24 women	Subcutaneous adipocytes	Low PPARγ expression levels
Mlinar et al. [93,94]	129 women	Subcutaneous adipocytes Visceral adipocytes	Positive correlation of lipin 1β expression in subcutaneous adipose tissue with *PPARγ*, Positive correlation of *HSD11B1* expression in visceral adipose tissue with *PPARγ*
Dumesic et al. [95]	16 women	Subcutaneous adipocytes	Positive prediction of total body mass, total body fat, and gynoid fat masses by *PPARγ* gene expression

**Table 3:** The influence of natural agents on PPAR expression in PCOS.

Natural Agent	Study Group Size	PPARα Upregulation	PPARγ Upregulation	Reference
Eugenol	30 rats	X		[96]
Icariin	36 rats	X		[97]
Labisia pumila standardized water extract	22 rats		X	[98]
γ-linolenic acid	Rat model		X	[99]
Fresh leaves extracts of Ficus religiosa	42 rats		X	[100]
Astragaloside IV	30 rats		X	[101]
Myo-inositol	45 rats; 53 women		X	[102,115]
Vitamin D3	Mouse model		X	[103]
Eicosapentaenoic acid	30 women		X	[104]
ω-3 fatty acids +/− vitamin E	100 women		X	[105,106]
Curcumin	132 women		X	[107,108]
Chromium +/− carnitine	94 women		X	[109,110]
Selenium	40 women		X	[111]
Coenzyme Q10	40 women		X	[112]
Fish oil	40 women		X	[113]
Melatonin	58 women		X	[114]

## Data Availability

Not applicable.
